# Cleft lip Sidedness and the Association with Additional Congenital Malformations

**DOI:** 10.1177/10556656241261918

**Published:** 2024-06-13

**Authors:** Matthew Fell, Kate J. Fitzsimons, Mark J. Hamilton, Jibby Medina, Sophie Butterworth, Min Hae Park, Jan Van der Meulen, Sarah Lewis, David Chong, Craig JH Russell

**Affiliations:** 1 Spires Cleft Centre, John Radcliffe Hospital, Oxford, UK; 2 The Cleft Collective, 12202 Bristol Dental School, 1980 University of Bristol, Bristol, UK; 3 Cleft Registry and Audit Network, Clinical Effectiveness Unit, 14211 The Royal College of Surgeons of England, London UK; 4 West of Scotland Centre for Genomic Medicine, 473300 Queen Elizabeth University Hospital, Glasgow UK; 5 Department of Health Services Research and Policy, 4906 London School of Hygiene and Tropical Medicine, London UK; 6 59842 The Royal Children's Hospital, Melbourne, Australia; 7 34361 Royal Hospital for Children, 473300 Queen Elizabeth University Hospital, Glasgow, UK

**Keywords:** cleft lip, cleft lip and palate, epidemiology, etiology

## Abstract

**Objective:**

To investigate the association between the sidedness of orofacial clefts and additional congenital malformations.

**Design:**

Linkage of a national registry of cleft births to national administrative data of hospital admissions

**Setting:**

National Health Service, England

**Participants:**

2007 children born with cleft lip ± alveolus (CL ± A) and 2724 with cleft lip and palate (CLP) born between 2000 and 2012.

**Main outcome measure:**

The proportion of children with ICD-10 codes for additional congenital malformations by the sidedness (left, right or bilateral) of orofacial clefts.

**Results:**

For CL ± A phenotypes, there was no evidence for a difference in the prevalence of additional anomalies between left (22%, reference), right (22%, aOR 1.02, 95% CI 0.80 to 1.28; *P* = .90) and bilateral clefts (23%, aOR 1.09, 95% CI 0.75 to 1.57; *P* = .66). For CLP phenotypes, there was evidence of a lower prevalence of additional malformations in left (23%, reference) compared to right (32%, aOR 1.54, 95% CI 1.25 to 1.91; *P* < .001) and bilateral clefts (33%, aOR 1.64, 95% CI 1.35 to 1.99; *P* < .001).

**Conclusions:**

The prevalence of additional congenital malformations was similar across sidedness subtypes with CL ± A phenotypes but was different for sidedness subtypes within CLP cases. These data support the hypothesis that CL ± A has a different underlying aetiology from CLP and that within the CLP phenotype, right sided CLP may lie closer in aetiology to bilateral CLP than it does to left sided CLP.

## Background

There are consistent trends for sidedness in clefts of the lip with or without palate (CL ± P) that has been termed ‘directional asymmetry’.^1,2^ The prevalence of left unilateral CL ± P is twice that of right unilateral CL ± P.^2,3^ Bilateral CL ± P occurs less commonly than unilateral CL ± P, with a quoted prevalence ratio of 6:3:1 for left/right/bilateral sidedness, respectively.^4^ Directional asymmetry is not a unique phenomenon to CL ± P, as left sided predominance is also observed in preauricular skin tags, congenital hip dysplasia and absent upper limb, whereas right sided predominance is seen in microtia and pre-auricular sinus.^5^ Both genetic and environmental factors have been proposed in the aetiology of sidedness in CL ± P, yet mechanisms are currently poorly understood.^6^

Orofacial clefts can occur in isolation or alongside additional congenital malformations (ACMs). Congenital malformations have been defined as defects involving a functional, structural, morphological or positional anomaly of a whole or part of a body organ system that tends to be macroscopic and occurs before birth.^7^ ACMs can occur as part of a syndrome, which may arise due to a change within the genetic material (including single-gene disorders or larger chromosome anomalies), teratogenic exposures or disruption events during development.^8^ However, the distinction between syndromic and non-syndromic clefts may not always be readily apparent at cleft diagnosis. Presently, consensus guidance is lacking both with regard to screening for additional malformations and genetic investigation for children born with orofacial clefts. The study and identification of co-occurring ACMs is therefore illuminating because it can give insights into the underlying aetiology of orofacial clefts,^9^ and helps to inform the design of bespoke management pathways.^10^

The United Kingdom (UK) is well positioned to investigate the association of CL ± P sidedness with ACMs due to the systematic collection of data on cleft at a nationwide level within a centralised healthcare system, which is free at the point of access. We have previously published the range and frequency of ACMs in 9403 children born with orofacial clefts and noted a difference in prevalence of ACMs between cleft lips ±  alveolus (CL ± A) and cleft lip and palate (CLP).^11^ This study aims to investigate whether there is any difference in the prevalence of ACMs by sidedness in both CL ± A and CLP using two national datasets in England, linked at the individual patient level.

## Methods

### Data Source

The study cohort was identified in the Cleft Registry and Audit NEtwork (CRANE) Database which is a national registry of all live-born children with an orofacial cleft (OFC) in the UK. Cases registered are most commonly identified antenatally or at birth (84%) and overall case ascertainment has recently been calculated as above 95%.^12^ Children whose parents had given consent for their child's records to be linked to other datasets were eligible to be linked to the Hospital Episode Statistics (HES) database.

The HES database^13^ contains records on all diagnoses and treatments made and given during admissions to National Health Service (NHS) hospitals in England. HES is derived from routine submission of data from each individual NHS hospital admission, primarily for the purposes of payment for and commissioning of healthcare in England.^14^ Data is inputted locally by professional health coders before undergoing central data quality processing for validity and data cleaning checks prior to being published. The linked dataset contained records on births from January 1st 2000 up to December 31st 2012 and hospital admissions up to March 31st 2015, to allow sufficient time for the existence of ACMs to be identified and recorded.

### Cleft Subtype Classification

Children born with either a unilateral or bilateral cleft lip with or without a cleft palate were included. In CRANE, cleft phenotypes and sidedness are categorised according to the LAHSAL classification (see supplementary Table 1).^15^ In HES, cleft type was assigned according to the presence of selected procedure codes (Classification of Interventions and Procedures, OPCS 4.5) and/or diagnosis codes (tenth revision of the International Classification of Diseases, ICD-10)^16^ in any of the available HES records. A stepwise, hierarchical approach was employed. First, the cleft reconstruction procedure codes (F03, F29, F32) were used to identify cleft type groups: CL ± A, CLP and cleft palate only. Second, the diagnosis code was used to distinguish between unilateral and bilateral cases. Children with cleft palate only and those whose cleft type was discordant between the two data sources were excluded from the study cohort.

### Additional Congenital Malformations (ACMs)

ACMs were classified based on the International Classification of Disease Version 10 (ICD-10) codes^16^ which are reported in HES data. ICD-10 is considered the international standard diagnostic classification for disease and is developed and maintained by the World Health Organisation. Congenital malformations, deformations and chromosome abnormalities are coded Q00-Q99 and are categorised according to the body or organ system they affect (see Appendix 1). Codes for ACMs relating to cleft lip and palate (Q35-Q37) were excluded from the analysis. The code for nasal ACMs (Q30) was included in the analysis because despite the nose being positioned anatomically adjacent to the lip and palate, the specified nasal anomalies would not be expected to represent the underlying orofacial cleft (Q30.1 Agenesis and underdevelopment of nose; Q30.2 Fissured, notched and cleft nose; Q30.3 Congenital perforated nasal septum; Q30.8 Other congenital malformations of nose; Q30.9 Congenital malformation of nose, unspecified). Similarly, code Q38 relating to malformations of tongue, mouth and pharynx was included as it specified that it excluded cleft lip and/or palate.

We did not attempt to remove individuals with a specific genetic and/or syndromic diagnosis from the analysis cohort, since there was no means of reliably achieving this distinction using ICD-10 codes.

### Data Analysis

The proportion of children with ICD-10 codes for congenital malformations (listed in Appendix 1) was examined. Differences in the percentage of children with ACMs between sidedness subgroups were initially explored using the Pearson chi-square test of independence. Children born with CL ± A were analysed separately from children born with CLP, as previous findings indicated different proportions of ACMs between the two phenotypes,^11^ and in line with developing knowledge to suggest that these cleft subtypes should be considered as distinct aetiological entities.^17–20^

As an additional analysis, the number of different body or organ systems with malformations was summed for each child, and the percentage of children with ACMs affecting two or more body systems was reported by cleft subtype. ICD-10 codes Q90-Q99 (Chromosomal abnormalities not elsewhere specified) were not counted in this additional analysis as they do not relate to a particular body system.

The association between cleft sidedness (left, right or bilateral) and the presence of ACMs was analysed using logistic regression to estimate effect size, using left sided clefts as a reference group and adjusting for patient sex. Additional adjustment was made by adding ethnicity into the regression model, but due to incomplete ethnicity data reducing the sample size, these results are reported as supplementary data only. Odds ratios, confidence intervals and p values are reported and interpreted as continuous measures of the strength of evidence against the null hypothesis.^21^ Statistical analysis was performed in Stata nV.17 (Statacorp).

### Ethical Considerations

This study is exempt from NHS Research Authority ethics approval as it involves the analysis of an existing anonymised dataset that is collected for the purpose of service evaluation.^22^

## Results

### Patient Characteristics

5940 CRANE-registered children were born alive in England with a CL ± P between 1st January 2000 and 31st December 2012. 5640 (95%) CRANE records were successfully linked to HES records but 1209 were excluded due to cleft phenotype discordance between the two data sources. The final cohort in this study included 4731 children born with CL ± P and of these, 42% had CL ± A and 58% had CLP (see Table 1). Among children with unilateral clefts, the ratio between left and right-sides was similar between those with CL ± A and those with CLP (65%:35% vs 63%:37% respectively, *P* = .279), However, the distribution of left, right and bilateral clefts differed between the CL ± A and CLP groups (59%, 32%, 9% vs. 43%, 25%, 32% respectively, *P* < .001).

**Table 1.** Characteristics of the Children Included in the Analyses and the Number and Percentage of Those with ≥1 Additional Congenital Malformations (ACM).

		Full study cohort		≥1 ACM	
Characteristic		N	(%)	n	(%)
Full study cohort		4731	100.0%	1212	25.6%
Type of cleft	Sidedness				
Cleft lip ± alveolus (CL ± A)	Total	2007	42.4%	435	21.7%
	Left	1183	25.0%	254	21.5%
	Right	636	13.4%	138	21.7%
	Bilateral	188	4.0%	43	22.9%
Cleft lip + palate (CLP)	Total	2724	57.6%	777	28.5%
	Left	1168	24.7%	271	23.2%
	Right	689	14.6%	219	31.8%
	Bilateral	867	18.3%	287	33.1%
Sex					
	Female	1608	34.0%	398	24.8%
	Male	3123	66.0%	814	26.1%
Ethnicity					
	Caucasian	3224	68.1%	801	24.8%
	Mixed	89	1.9%	34	38.2%
	Asian	263	5.6%	79	30.0%
	Black	63	1.3%	16	25.4%
	Other	99	2.1%	32	32.3%
	Unknown	993	21.0%	250	25.2%

66% of the cohort was male. This proportion differed between CL ± A and CLP groups (63% vs 68%, *P* < .001) but there was no difference in the proportion of ACMs between males and females (26% vs 25% *P* = .33). Among those with reported ethnicity (79%), the proportion identified as Caucasian was similar between the CL ± A and CLP groups (87% vs 86%, *P* = .58). The overall proportion of ACMs was lowest in Caucasian and Black ethnicities and highest in Mixed, Asian and ‘Other’ ethnicity groups (see Table 1).

### Additional Congenital Malformations (ACMS)

**Figure 1.**
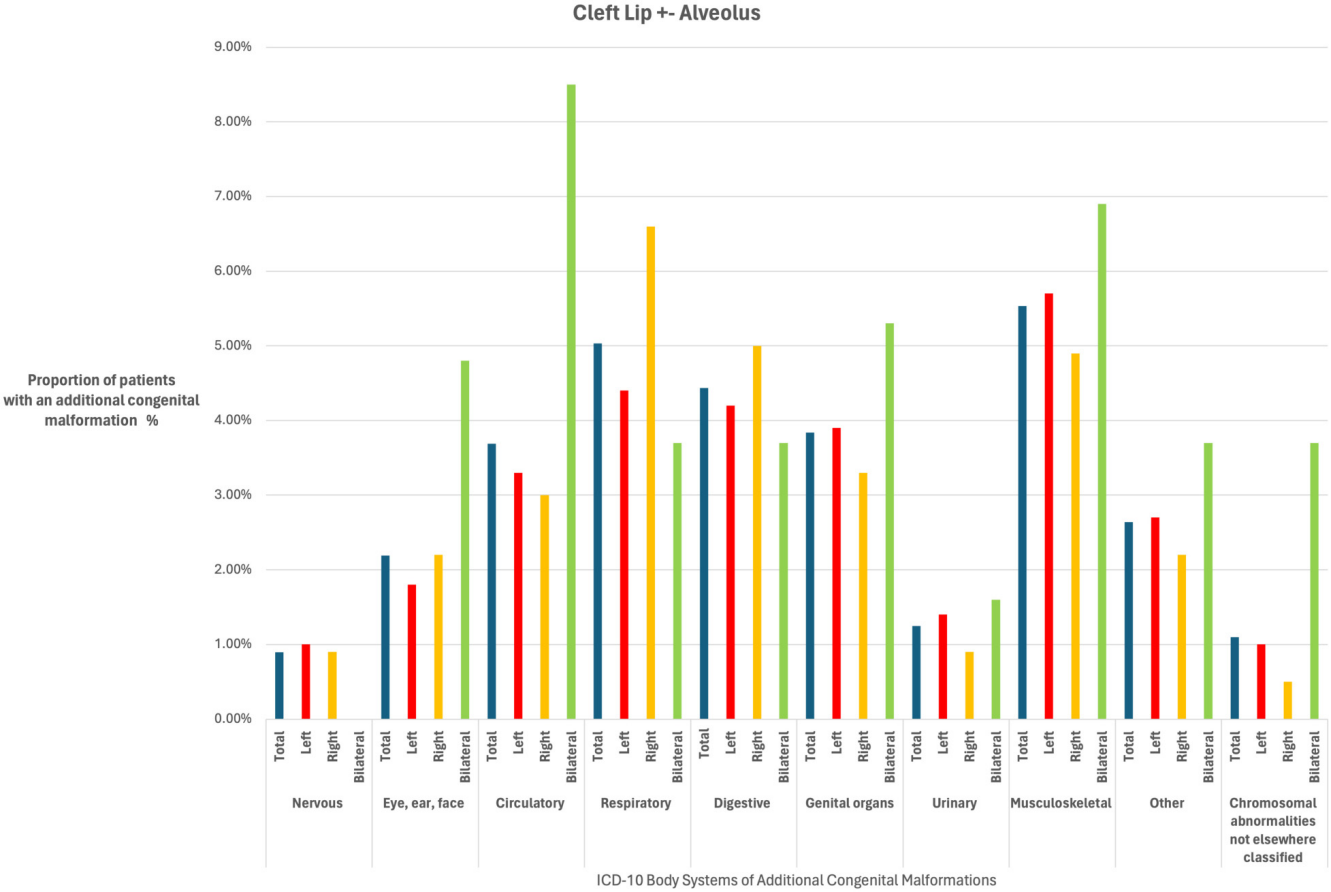
A bar chart to show the proportion (%) of patients born with a cleft lip ±  alveolus with additional congenital malformations according to cleft sidedness (left, right and bilateral) for body systems in the International Classification of Disease Version 10 (ICD-10) system.

**Figure 2.**
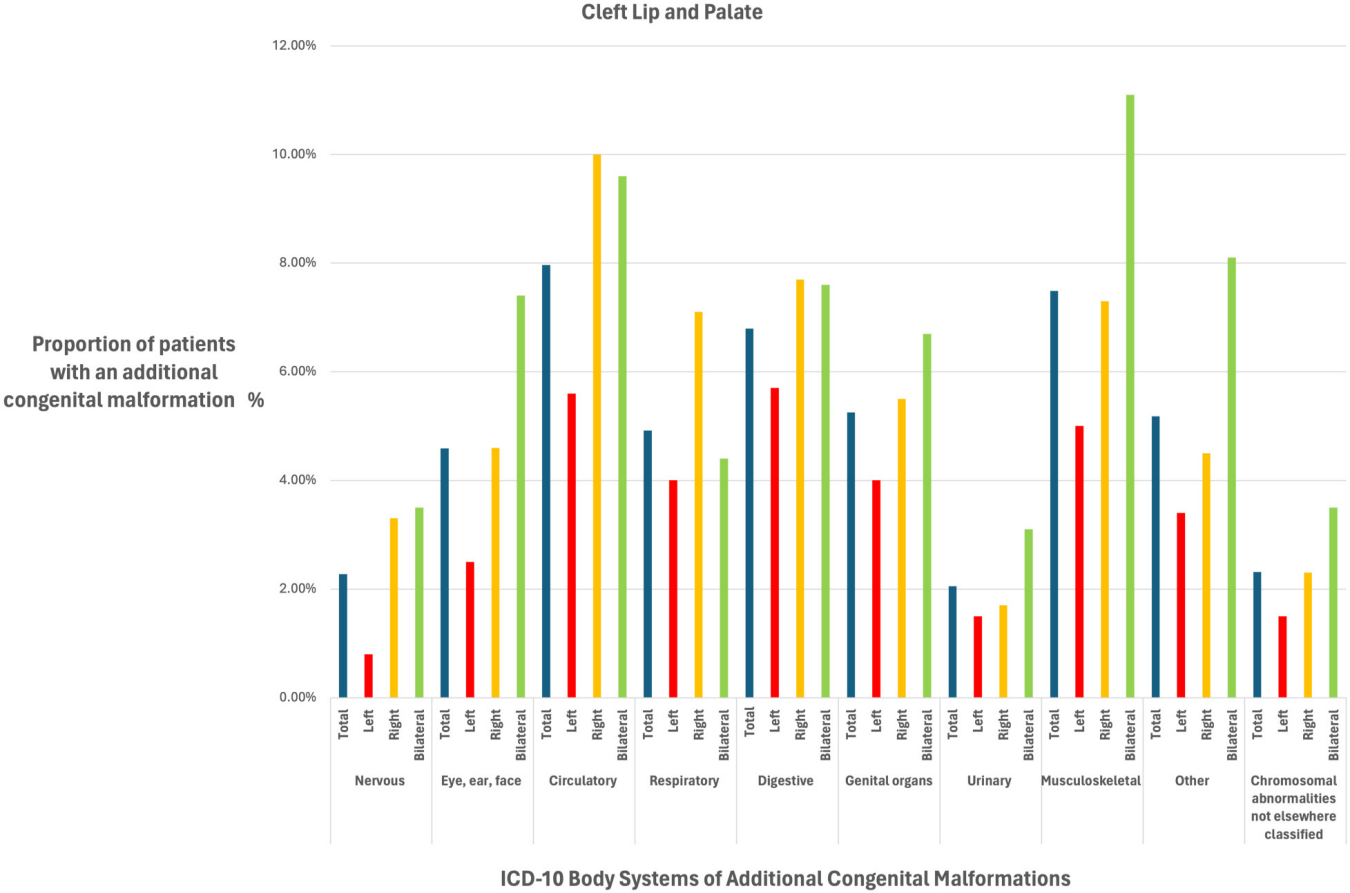
A bar chart to show the proportion (%) of patients born with a cleft lip + palate with additional congenital malformations according to cleft sidedness (left, right and bilateral) for body systems reported in the International Classification of Disease Version 10 (ICD-10) system.

The prevalence of ACMs for the different cleft subtypes and sidedness are detailed in Tables 2 and 3 and Figures 1 and 2. When categorised by body systems, the two most prevalent ACMs for the CL ± A cohort were musculoskeletal (5%) and respiratory (5%), whereas for the CLP cohort they were circulatory (8%) and musculoskeletal (8%).

**Table 2.** The Association of Additional Congenital Malformations (ACM), According to Cleft Type (Cleft lip ± Alveolus (CL ± A) and Cleft Lip and Palate (CLP)) and Sidedness. Effect Estimate is the Odds Ratio (OR) with 95% Confidence Intervals (Cis) and P Values.

Cleft Type	Sidedness	Total	≥1 ACM							
		N	N	(%)	OR	(95% CI)	*P* value	aOR*	(95% CI)	*P* value
CL ± A	Left	1183	254	21.5%	Reference			Reference		
	Right	636	138	21.7%	1.01	(0.80 to 1.28)	.80	1.02	(0.80 to 1.28)	.90
	Bilateral	188	43	22.9%	1.08	(0.75 to 1.57)	.75	1.09	(0.75 to 1.57)	.66
CLP	Left	1168	271	23.2%	Reference			Reference		
	Right	689	219	31.8%	1.54	(1.25 to 1.90)	<.001	1.54	(1.25 to 1.91)	<.001
	Bilateral	867	287	33.1%	1.64	(1.35 to 1.99)	<.001	1.64	(1.35 to 1.99)	<.001

* Adjusted for sex.

**Table 3.** The Association of Additional Congenital Malformations (ACM) According to ICD-10 Body System Classification, by Cleft Type (Cleft lip ± Alveolus (CL ± A) and Cleft Lip and Palate (CLP)) and Sidedness. Effect Estimate is the Odds Ratio (OR) with 95% Confidence Intervals (Cis) and *P* Values.

ICD-10 Code	Body System	Sidedness	CL ± A						CLP					
			Total	≥1 ACM					Total	≥1 ACM				
			N	n	%	OR	(95% CI)	*P* value	N	n	%	OR	(95% CI)	*P* value
Q00-Q07	Nervous	Left	1183	12	1.0%	Reference			1168	9	0.8%	Reference		
		Right	636	6	0.9%	0.93	(0.35 to 2.49)	.88	689	23	3.3%	4.45	(2.05 to 9.67)	<.001
		Bilateral	188	0	0.0%	1	-	-	867	30	3.5%	4.62	(2.18 to 9.77)	<.001
Q10-Q18	Eye, ear, face	Left	1183	21	1.8%	Reference				29	2.5%	Reference		
		Right	636	14	2.2%	1.25	(0.63 to 2.47)	.53	689	32	4.6%	1.91	(1.15 to 3.19)	.01
		Bilateral	188	9	4.8%	2.78	(1.25 to 6.17)	.01	867	64	7.4%	3.13	(2.00 to 4.90)	<.001
Q20-Q28	Circulatory	Left	1183	39	3.3%	Reference				65	5.6%	Reference		
		Right	636	19	3.0%	0.90	(0.52 to 1.58)	.72	689	69	10.0%	1.89	(1.33 to 2.69)	<.001
		Bilateral	188	16	8.5%	2.73	(1.49 to 4.99)	.001	867	83	9.6%	1.8	(1.28 to 2.52)	<.001
Q30-Q34	Respiratory	Left	1183	52	4.4%	Reference				47	4.0%	Reference		
		Right	636	42	6.6%	1.54	(1.01 to 2.34)	.04	689	49	7.1%	1.83	(1.21 to 2.76)	.004
		Bilateral	188	7	3.7%	0.84	(0.38 to 1.89)	.67	867	38	4.4%	1.09	(0.71 to 1.69)	.69
Q38-Q45	Digestive	Left	1183	50	4.2%	Reference				66	5.7%	Reference		
		Right	636	32	5.0%	1.2	(0.76 to 1.89)	.43	689	53	7.7%	1.39	(0.96 to 2.02)	.08
		Bilateral	188	7	3.7%	0.88	(0.39 to 1.96)	.75	867	66	7.6%	1.38	(0.97 to 1.96)	.08
Q50-Q56	Genital organs	Left	1183	46	3.9%	Reference				47	4.0%	Reference		
		Right	636	21	3.3%	0.84	(0.50 to 1.43)	.53	689	38	5.5%	1.39	(0.90 to 2.16)	.14
		Bilateral	188	10	5.3%	1.39	(0.69 to 2.80)	.36	867	58	6.7%	1.71	(1.15 to 2.54)	.007
Q60-Q64	Urinary	Left	1183	16	1.4%	Reference				17	1.5%	Reference		
		Right	636	6	0.9%	0.69	(0.27 to 1.78)	.45	689	12	1.7%	1.2	(0.57 to 2.53)	.63
		Bilateral	188	3	1.6%	1.18	(0.34 to 4.10)	.79	867	27	3.1%	2.18	(1.18 to 4.02)	.01
Q65-Q79	Musculoskeletal	Left	1183	67	5.7%	Reference				58	5.0%	Reference		
		Right	636	31	4.9%	0.85	(0.55 to 1.32)	.48	689	50	7.3%	1.5	(1.01 to 2.21)	.04
		Bilateral	188	13	6.9%	1.24	(0.67 to 2.29)	.50	867	96	11.1%	2.38	(1.70 to 3.34)	<.001
Q80-Q89	Other	Left	1183	32	2.7%	Reference				40	3.4%	Reference		
		Right	636	14	2.2%	0.81	(0.43 to 1.52)	.51	689	31	4.5%	1.33	(0.92 to 2.14)	.24
		Bilateral	188	7	3.7%	1.39	(0.60 to 3.20)	.44	867	70	8.1%	2.48	(1.66 to 3.69)	<.001
Q90-Q99	Chromosomal abnormalities not elsewhere classified	Left	1183	12	1.0%	Reference				17	1.5%	Reference		
		Right	636	3	0.5%	0.46	(0.13 to 1.64)	.23	689	16	2.3%	1.61	(0.81 to 3.21)	.18
		Bilateral	188	7	3.7%	3.77	(1.47 to 9.71)	.006	867	30	3.5%	2.43	(1.33 to 4.43)	.004

### Cleft Lip ±  Alveolus (CL ± A)

When compared to the proportion of ACMs in left sided CL ± A (22%), there was no difference in the proportion of ACMs in either right UCL ± A (22%, aOR 1.02, 95% CI 0.80 to 1.28; *P* = .90) or bilateral UCL ± A (23%, aOR 1.09, 95% CI 0.75 to 1.57; *P* = .66) as seen in Table 2. Additional adjustment for ethnicity did not alter this finding (see Supplementary Table 2).

When ACMs were stratified by the ICD-10 body system subgroups, the ten subcategories were present in similar proportions among children with a left, right or bilateral CL ± A in many of the body systems (See Table 3). When compared to the left CL ± A reference group, respiratory anomalies were more common in children with a right CL ± A (OR 1.54, 95% CI 1.01 to 2.34; *P* = .04), whereas the bilateral CL ± A phenotype had a higher proportion of eye, ear, face anomalies (OR 2.78, 95% CI 1.25 to 6.17; *P* = .01) and circulatory anomalies (OR 2.73, 95% CI 1.49 to 4.99; *P* = .001), although the evidence for these associations was weak given that we performed multiple tests.

### Cleft Lip and Palate (CLP)

When compared to the proportion of ACMs in left sided CLP (23%) there was a higher proportion of ACMs amongst both right CLP (32%, aOR 1.54, 95% CI 1.25 to 1.91; *P* < .001) and bilateral CLP (33%, aOR 1.64, 95% CI 1.35 to 1.99; *P* < .001) as seen in Table 2. Additional adjustment for ethnicity did not alter this trend (Supplementary Table 2).

When ACMs were stratified by the ICD-10 body system subgroups, each of the ten subcategories of ACMs were more prevalent in both right and bilateral CLP when compared to the left CLP reference (see Table 3) The direction of the effect estimate for right and bilateral clefts was consistent for each body system, but the strength of association for ACMs compared to left CLP varied depending on the body system.

### Number of Systems Affected by Additional Congenital Malformations

Table 4 shows the number and percentage of children who had multiple (≥2) body systems (eg, nervous, eye/ear/face, circulatory, respiratory, digestive, genital, urinary and musculoskeletal systems) affected by ACMs. Among those with CL ± A, the proportion of children who had malformations across multiple body systems when compared to left CL ± A (5%) was similar for right CL ± A (4%, aOR 0.74, 95% CI 0.44 to 1.22; *P* = .23) but was greater for bilateral CL ± A (10%, aOR 2.17, 95% CI 1.25 to 3.79; *P* < .001). Among those with CLP, the proportion of children who had ACMs across multiple body systems when compared to left CLP (6%), was greater for both right CLP (11%, aOR 1.76, 95% CI 1.25 to 2.46; *P* < .001) and bilateral CLP (14%, aOR 2.45, 95% CI 1.81 to 3.31; *P* < .01).

**Table 4.** Multiple (≥2) Body Systems Affected by Additional Congenital Malformations (ACM), According to Cleft Type (Cleft lip ± Alveolus (CL ± A) and Cleft lip and Palate (CLP)) and Sidedness. Effect Estimate is the Odds Ratio (OR) with 95% Confidence Intervals (Cis) and *P* Values.

Cleft Type	Sidedness	Total	≥2 body systems with ≥1 ACM							
		N	n	(%)	OR	(95% CI)	*P* value	aOR*	(95% CI)	*P* value
CL ± A	Left	1183	55	4.6%	Reference			Reference		
	Right	636	22	3.5%	0.73	(0.44 to 1.22)	.23	0.74	(0.44 to 1.22)	.23
	Bilateral	188	18	9.6%	2.17	(1.25 to 3.79)	.006	2.17	(1.25 to 3.79)	.006
CLP	Left	1168	74	6.3%	Reference			Reference		
	Right	689	73	10.6%	1.75	(1.25 to 2.46)	.001	1.76	(1.25 to 2.46)	.001
	Bilateral	867	123	14.2%	2.44	(1.81 to 3.31)	<.001	2.45	(1.81 to 3.31)	<.001

* Adjusted for sex.

## Discussion

### Summary of Findings

In this national sample of 4731 children born with CL ± P, we were able to examine the association of cleft sidedness with the prevalence of ACM as coded by the ICD-10 classification. The prevalence of ACMs for children born with CL ± A ranged between 22% to 23% and whilst there was no evidence to suggest differences between left, right or bilateral sidedness, there was evidence to suggest a greater proportion of ACMs in multiple body systems in bilateral CL ± A (10%) compared to left (5%) or right (4%) CL ± A.

The prevalence of ACMs for children born with CLP was different according to sidedness, with 23% of left sided CLP having at least one additional malformation compared to 32% in right CLP and 33% in bilateral CLP. The subcategorization of ICD-10 malformation codes into body systems within the CLP subtype, consistently showed a higher prevalence of malformations for right and bilateral CLP compared to left CLP in each body system, reducing the likelihood that this trend is a chance finding. In addition, the proportion of ACMs in multiple body systems was greater for both right (11%) and bilateral (14%) CLP compared to left CLP (6%).

### Comparison to Previous Studies

Pereira et al.,^10^ analysed the presence of ACMs in 701 children born with orofacial clefts from a Portuguese database, 343 of whom had unilateral CL ± P. Among children with ACMs, there was a higher prevalence of right CL ± P compared to the group without ACMs (13% vs 11%; *P* < .001). The authors did not stratify individual ACMs by sidedness but, for the entire cohort, head and neck anomalies were found to be the most common, followed by cardiovascular anomalies.

Gallagher et al.,^23^ reported a greater prevalence of syndromes amongst 155 children born with a right sided CL ± P compared to 276 born with a left sided CL ± P (OR 1.9, 95% CI 0.7 to 4.7). The imprecise estimate may have been linked to the small sample size and subjective method of identifying syndromes. The authors concluded that children born with right sided CL ± P were more likely to be born with ACMs and whilst this is consistent with our data, they did not subcategorise the clefts into CL ± A and CLP subtypes.

### Interpretation of the Data

Our data can be used to consider the current theories that have been suggested for the aetiology of sidedness in CL ± P. Various case control and genome wide association studies (GWAS) have highlighted distinct genetic variations associated with left versus right sided CL ± P over the past two decades.^20,24–28^ For example, using a GWAS approach, Curtis et al.,^20^ found evidence that a polymorphism at locus 4q28, close to the *FAT4* gene, influenced sidedness in unilateral CL ± A, whilst no significant effect was detected for this locus in unilateral CLP. For decades, since pioneering work by Fogh-Anderson,^29^ it has been recognised that the aetiology of cleft palate only is likely different to that of CL ± P. Further, subsequent evidence has emerged to suggest distinct aetiologies between CL ± A and CLP.^17^ Our data contributes further evidence that sidedness may also be distinct between CL ± A and CLP subtypes.

The bilateral CL ± P cohort in this study provides a useful comparative group for the unilateral CL ± P cohort. First, cleft lip sidedness ratios are often quoted in the literature as 6:3:1 for left:right:bilateral^4^ and whilst this was true in our CL ± A cohort, the proportion of bilateral CLP was similar to right CLP, as has been previously reported in other orofacial cleft epidemiology studies.^2,30^ Second, for the CL ± A subtype, the prevalence of ACMs is similar irrespective of left, right or bilateral sidedness, whereas for the CLP subtype the prevalence of ACMS is higher for right and bilateral CLP compared to left CLP.

**Figure 3.**
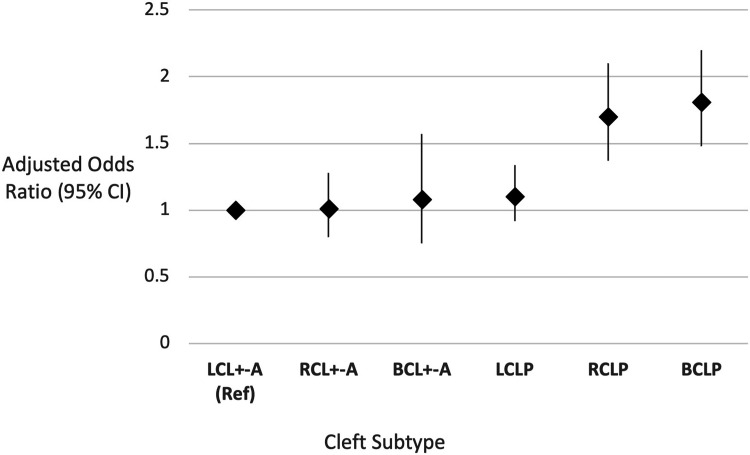
The association of additional congenital malformations across the spectrum of unilateral and bilateral cleft lip with or without cleft palate. Groups are categorised according to sidedness left (L), right (R) and bilateral (B) and the cleft subtypes cleft lip ±  alveolus (CL ± A) and cleft lip and palate (CLP). Left unilateral cleft lip ± A (LUCL ± A) is used as the reference group. Effect estimates displayed as Odds Ratio (OR) with 95% Confidence Intervals (CI), adjusted for sex.

The amalgamation of all cleft subtypes included in our main and supplementary analysis can be presented as a spectrum using left CL ± A as a reference (see Figure 3 and supplementary Table 3) and demonstrates right and bilateral CLP standing apart from the other cleft lip subtypes, according to the proportion of associated ACMs. In two studies investigating the co-occurrence of congenital dental anomalies with cleft sidedness, there was a reported higher prevalence of left maxillary lateral incisor agenesis in right CL/P compared with the reverse scenario.^31,32^ Both authors suggested the occurrence of a missing lateral incisor on the non-cleft side may represent a bilateral genotype that did not fully manifest and that right sided CL ± P may be more likely to correspond to a milder phenotype of bilateral clefts.^33,34^

Investigations into the aetiology of orofacial cleft have reached a broad consensus that non-syndromic clefts arise within a multifactorial background.^33,34^ According to the Multifactorial threshold Model of Inheritance, right sided CLP and bilateral CLP occur less commonly than left sided CLP, requiring a higher threshold of genetic and environmental influences for them to occur and may represent more severe phenotypes.

### Strengths and Limitations

This is a large cohort of standardised national data within a centralised and nationalised health care system, and therefore represents a unique resource. The CRANE registry has high case ascertainment,^12^ and given its national coverage is likely to be representative of children born with an orofacial in England during the study period. A minimum follow-up of 2.25 years in HES data allowed sufficient time for congenital anomalies to be identified after birth. Although the cohort was born between 2000 and 2012, we believe that the knowledge gained from this study is still relevant to people born with an orofacial cleft in the present day. Discordance in cleft subtypes in one fifth of the patient records between the datasets was a weakness as it resulted in the exclusion of otherwise successfully linked data.

The ICD-10 categorisation system of malformations is advantageous because it is an international system that is widely accessible and facilitates thorough documentation of anatomical detail. However, despite the ICD-10 being regarded as the international standard, a variety of different classifications have been used in the literature, which makes comparison difficult.^10,35^ The ICD-10 anatomical subcategorization is not necessarily clinically intuitive and cannot differentiate between minor and major ACMs. Whilst the use of ICD-10 codes allowed us to report many congenital malformations, HES restricts the entry of these codes to 4 characters (eg, Q87.0), which means that some codes were not sensitive enough to distinguish between certain diagnoses (for example, Pierre Robin Sequence and Goldenhar syndrome share the same 4-character ICD-10 code). Furthermore, ICD-10 codes utilised in HES tend to focus on a physical diagnosis or phenotype, rather than the underlying genetic cause. This means the prevalence of specific genetic and/or syndromic diagnoses associated with orofacial clefts and other congenital malformations could not be reported. With the now widespread application of genomic testing, this limitation highlights a need for cleft research cohorts to accurately capture data relating to genetic and syndromic diagnoses.

This study was restricted to children born alive. It was not possible to include spontaneous abortions, elective terminations and stillborn foetuses. Furthermore, as there is no standard protocol for evaluating other body systems for anomalies in children presenting with a cleft in England, there may well be subclinical and untreated anomalies that have been missed in the study population. Whilst there is no reason to suggest that the prevalence of missed ACMs would differ according to cleft sidedness, the true prevalence of ACMs is likely to be underrepresented.

Finally, we adjusted for the potential confounders of sex and ethnicity as these variables were readily available in the data set. Adjustment for these confounders did not alter the trends observed indicating that the reported findings were not explained by these characteristics. The proportion of children with additional malformations according to ethnic background was limited by missing data and slightly lower representation of minority ethnic groups in this cohort as compared to last UK census.^36^ Furthermore, we were not able to add additional confounding factors to our model, which could be important in cleft aetiology, such as maternal age, Body Mass Index, and folic acid consumption.

### Further Work

Our study demonstrates the importance of any study on cleft sidedness being explicit about the inclusion or exclusion of children born with ACMs, as the proportion of ACMs will likely be different for children born with left, right and bilateral CLP. Further work to understand the aetiology of sidedness in CL ± P should stratify participants by CL ± A and CLP subtypes. Furthermore, future studies should make efforts to distinguish individuals within cleft cohorts who have a known monogenic or chromosomal diagnosis, from those whose cleft is assumed to have a complex or multifactorial cause, since genetic influences may be overlapping or distinct between these groups.

Our results suggest a research focus on left CLP versus right and bilateral CLP is warranted. Work to investigate genetic factors should be expanded to include large scale GWAS studies. DNA methylation has been shown to play a critical role in establishing laterality of the body plan during early embryogenesis in zebrafish models,^37^ and methylation patterns have been shown to differ by CL ± A, CLP and CP subtype in humans,^17^ yet we are not aware of its use in orofacial cleft sidedness research and studying methylation patterns could offer important insights in this area. This may help to enhance our understanding of how cleft sub-phenotypes are classified and provide more information to support or refute the suggestion of right sided CLP and bilateral CLP being different phenotypic expressions of the same condition.

## Conclusion

This large population-based study provides evidence for an increased risk of ACMs among right and bilateral CLP compared to left CLP. This was not the case for CL ± A, where ACMs were equivalent according to cleft sidedness. This supports a growing evidence base to suggest CLP has a different aetiology when compared with CL ± A and that these subtypes of cleft should not be considered as a single entity. Furthermore, this data suggests that right CLP may lie closer in aetiology to bilateral CLP than it does to left sided CLP. Our findings may provide further insights into why right sided orofacial clefts are less prevalent than left sided orofacial clefts, although further work is required to explain, define and understand this.

## Supplemental Material

sj-docx-1-cpc-10.1177_10556656241261918 - Supplemental material for Cleft lip Sidedness and the Association with Additional Congenital Malformations Supplemental material, sj-docx-1-cpc-10.1177_10556656241261918 for Cleft lip Sidedness and the Association with Additional Congenital Malformations by Matthew Fell, Kate J. Fitzsimons, Mark J. Hamilton, Jibby Medina, Sophie Butterworth, Min Hae Park, Jan Van der Meulen, Sarah Lewis, David Chong and Craig JH Russell in The Cleft Palate Craniofacial Journal

sj-docx-2-cpc-10.1177_10556656241261918 - Supplemental material for Cleft lip Sidedness and the Association with Additional Congenital Malformations Supplemental material, sj-docx-2-cpc-10.1177_10556656241261918 for Cleft lip Sidedness and the Association with Additional Congenital Malformations by Matthew Fell, Kate J. Fitzsimons, Mark J. Hamilton, Jibby Medina, Sophie Butterworth, Min Hae Park, Jan Van der Meulen, Sarah Lewis, David Chong and Craig JH Russell in The Cleft Palate Craniofacial Journal

sj-docx-3-cpc-10.1177_10556656241261918 - Supplemental material for Cleft lip Sidedness and the Association with Additional Congenital Malformations Supplemental material, sj-docx-3-cpc-10.1177_10556656241261918 for Cleft lip Sidedness and the Association with Additional Congenital Malformations by Matthew Fell, Kate J. Fitzsimons, Mark J. Hamilton, Jibby Medina, Sophie Butterworth, Min Hae Park, Jan Van der Meulen, Sarah Lewis, David Chong and Craig JH Russell in The Cleft Palate Craniofacial Journal

## Appendix 1: International Classification of Disease Version 10 (ICD-10) diagnostic codes used to identify congenital malformations in Hospital Episode Statistics.

**Table:**

ICD-10 code	Description
	***Congenital malformations of the nervous system***
Q00	Anencephaly and similar malformations
Q01	Encephalocele
Q02	Microcephaly
Q03	Congenital hydrocephalus
Q04	Other congenital malformations of brain
Q05	Spina bifida
Q06	Other congenital malformations of spinal cord
Q07	Other congenital malformations of nervous system
	***Congenital malformations of eye, ear, face and neck***
Q10	Congenital ptosis
Q11	Anophthalmos, microphthalmos and macrophthalmos
Q12	Congenital lens malformations
Q13	Congenital malformations of anterior segment of eye
Q14	Congenital malformations of posterior segment of eye
Q15	Other congenital malformations of eye
Q16	Congenital malformations of ear causing impairment of hearing
Q17	Other congenital malformations of ear
Q18	Other congenital malformations of face and neck
	***Congenital malformations of the circulatory system***
Q20	Congenital malformations of cardiac chambers and connections
Q21	Congenital malformations of cardiac septa
Q22	Congenital malformations of pulmonary and tricuspid valves
Q23	Congenital malformations of aortic and mitral valves
Q24	Other congenital malformations of heart
Q25	Congenital malformations of great arteries
Q26	Congenital malformations of great veins
Q27	Other congenital malformations of peripheral vascular system
Q28	Other congenital malformations of circulatory system
	***Congenital malformations of the respiratory system***
Q30	Congenital malformations of nose
Q31	Congenital malformations of larynx
Q32	Congenital malformations of trachea and bronchus
Q33	Congenital malformations of lung
Q34	Other congenital malformations of respiratory system
	***Other congenital malformations of the digestive system***
Q38	Other congenital malformations of tongue, mouth and pharynx
Q39	Congenital malformations of oesophagus
Q40	Other congenital malformations of upper alimentary tract
Q41	Congenital absence, atresia and stenosis of small intestine
Q42	Congenital absence, atresia and stenosis of large intestine
Q43	Other congenital malformations of intestine
Q44	Congenital malformations of gallbladder, bile ducts and liver
Q45	Other congenital malformations of digestive system
	***Congenital malformations of the genital organs***
Q50	Congenital malformations of ovaries, fallopian tubes and broad ligaments
Q51	Congenital malformations of uterus and cervix
Q52	Other congenital malformations of female genitalia
Q53	Undescended testicle
Q54	Hypospadias
Q55	Other congenital malformations of male genital organs
Q56	Indeterminate sex and pseudohermaphroditism
	***Congenital malformations of the urinary system***
Q60	Renal agenesis and other reduction defects of kidney
Q61	Cystic kidney disease
Q62	Congenital obstructive defects of renal pelvis and congenital malformations of ureter
Q63	Other congenital malformations of kidney
Q64	Other congenital malformations of urinary system
	***Congenital malformations and deformations of the musculoskeletal system***
Q65	Congenital deformities of hip
Q66	Congenital deformities of feet
Q67	Congenital musculoskeletal deformities of head, face, spine and chest
Q68	Other congenital musculoskeletal deformities
Q69	Polydactyly
Q70	Syndactyly
Q71	Reduction defects of upper limb
Q72	Reduction defects of lower limb
Q73	Reduction defects of unspecified limb
Q74	Other congenital malformations of limb(s)
Q75	Other congenital malformations of skull and face bones
Q76	Congenital malformations of spine and bony thorax
Q77	Osteochondrodysplasia with defects of growth of tubular bones and spine
Q78	Other osteochondrodysplasias
Q79	Congenital malformations of the musculoskeletal system, not elsewhere classified
	***Other congenital malformations***
Q80	Congenital ichthyosis
Q81	Epidermolysis bullosa
Q82	Other congenital malformations of skin
Q83	Congenital malformations of breast
Q84	Other congenital malformations of integument
Q85	Phakomatoses, not elsewhere classified
Q86	Congenital malformation syndromes due to known exogenous causes, not elsewhere classified
Q87	Other specified congenital malformation syndromes affecting multiple systems
Q89	Other congenital malformations, not elsewhere classified
	***Chromosomal abnormalities, not elsewhere classified***
Q90	Down syndrome
Q91	Edwards syndrome and Patau syndrome
Q92	Other trisomies and partial trisomies of the autosomes, not elsewhere classified
Q93	Monosomies and deletions from the autosomes, not elsewhere classified
Q95	Balanced rearrangements and structural markers, not elsewhere classified
Q96	Turner syndrome
Q97	Other sex chromosome abnormalities, female phenotype, not elsewhere classified
Q98	Other sex chromosome abnormalities, male phenotype, not elsewhere classified
Q99	Other chromosome abnormalities, not elsewhere classified

ICD-10, International Classification of Diseases – 10th Edition
