# Supplementary material for: Cleft lip Sidedness and the Association with Additional Congenital Malformations
Source: Cleft Palate Craniofac J. 2024 Jun 13;62(9):1504–15. doi: 10.1177/10556656241261918 (PMC12254516; doi:10.1177/10556656241261918)
Supplement: sj-docx-1-cpc-10.1177_10556656241261918 - Supplemental material for Cleft lip Sidedness and the Association with Additional Congenital Malformations [file sj-docx-1-cpc-10.1177_10556656241261918.docx]

**Supplementary Table 1:** Stratification by cleft phenotype was performed according to the LAHSAL (Lip, Alveolus, Hard palate, Soft palate, Alveolus, Lip) classification, adapted from the description by Kriens in 1987.^1^ We did not differentiate between incomplete and complete clefts in this study, therefore code for incompletes (denoted in the LAHSAL classification by non-capitalised letters) were included.

| **Cleft phenotype** | **LAHSAL Classification** |
| --- | --- |
| **LUCL+-A** (Left unilateral cleft lip with or without a cleft of alveolus) | …..L  ….AL |
| **RUCL+-A** (Right unilateral cleft lip with or without a cleft of alveolus) | L…..  LA…. |
| **BCL+-A** (Bilateral cleft lip with or without a cleft of alveolus) | L….L  LA…L  L…AL  LA..AL |
| **LUCLP** (Left unilateral cleft lip and palate) | ..HSAL  ..H,,L  ..H.AL  ..HS.L  …SAL  …S.L |
| **RUCLP** (Right unilateral cleft lip and palate) | LAHS..  LA.S..  LAH…  L..S..  L.H…  L.HS.. |
| **BCLP** (Bilateral cleft lip and palate) | LAHSHAL  L..S.L  L..SAL  L.H..L  L.HS.L  L.HSAL  LA.SAL  LAH..L  LAH.AL  LAHS.L |

1. Kriens O. Lahshal: A concise documentation system for cleft lip, alvolus and palate diagnoses. In: Kriens O, ed. *What Is Cleft Lip and Palate? A Multidisciplinary Update Workshop, Bremen*. Thieme; 1987.
