# Supplementary material for: Cleft lip Sidedness and the Association with Additional Congenital Malformations
Source: Cleft Palate Craniofac J. 2024 Jun 13;62(9):1504–15. doi: 10.1177/10556656241261918 (PMC12254516; doi:10.1177/10556656241261918)
Supplement: sj-docx-2-cpc-10.1177_10556656241261918 - Supplemental material for Cleft lip Sidedness and the Association with Additional Congenital Malformations [file sj-docx-2-cpc-10.1177_10556656241261918.docx]

**Supplementary Table 2.** Odds ratios (95% confidence intervals) for additional congenital malformations, according to cleft type and sidedness. Adjustment made for sex (male vs female) and ethnicity (White British vs BME). Reduced total numbers due to missing ethnicity data.

|  |  | | Total | ≥1 ACM | | | | | | | |
| --- | --- | --- | --- | --- | --- | --- | --- | --- | --- | --- | --- |
| Cleft type | | Sidedness | N | n | % | OR | (95% CI) | p value | aOR* | (95% CI) | p value |
| CL±A | | Left | 922 | 192 | 20.8% | Reference |  |  | Reference |  |  |
|  | | Right | 504 | 111 | 22.0% | 1.07 | (0.82 to 1.40) | 0.60 | 1.01 | (0.80 to 1.28) | 0.91 |
|  | | Bilateral | 150 | 36 | 24.0% | 1.2 | (0.80 to 1.80) | 0.38 | 1.07 | (0.74 to 1.54) | 0.73 |
| CLP | | Left | 934 | 217 | 23.2% | Reference |  |  | Reference |  |  |
|  | | Right | 553 | 177 | 32.0% | 1.56 | (1.23 to 1.97) | <0.001 | 1.57 | (1.24 to 1.99) | <0.001 |
|  | | Bilateral | 675 | 229 | 33.9% | 1.7 | (1.36 to 2.11) | <0.001 | 1.72 | (1.38 to 2.14) | <0.001 |

*Adjusted for sex and ethnicity
