# Supplementary material for: Cleft lip Sidedness and the Association with Additional Congenital Malformations
Source: Cleft Palate Craniofac J. 2024 Jun 13;62(9):1504–15. doi: 10.1177/10556656241261918 (PMC12254516; doi:10.1177/10556656241261918)
Supplement: sj-docx-3-cpc-10.1177_10556656241261918 - Supplemental material for Cleft lip Sidedness and the Association with Additional Congenital Malformations [file sj-docx-3-cpc-10.1177_10556656241261918.docx]

**Supplementary Table 3.** Proportion of additional congenital malformations across of the spectrum of unilateral and bilateral cleft lip with or without cleft palate using left UCL±A as the reference. Adjusted OR and confidence intervals used for Figure 1.

| **Cleft type & sidedness** | Total | | ≥1 ACM | | | | | | | | | | |
| --- | --- | --- | --- | --- | --- | --- | --- | --- | --- | --- | --- | --- | --- |
|  | N | | N | | (%) | | OR | | (95% CI) | p value | aOR* | (95% CI) | p value |
| LCL±A | 1,183 | | 254 | | 21.5% | | Reference | |  |  | Reference |  |  |
| RCL±A | 636 | | 138 | | 21.7% | | 1.01 | | (0.80 to 1.28) | 0.91 | 1.01 | (0.80 to 1.28) | 0.91 |
| BCL±A | 188 | | 43 | | 22.9% | | 1.08 | | (0.75 to 1.57) | 0.66 | 1.08 | (0.75 to 1.57) | 0.66 |
| LUCLP | 1,168 | | 271 | | 23.2% | | 1.10 | | (0.91 to 1.34) | 0.31 | 1.10 | (0.91 to 1.34) | 0.32 |
| RUCLP | 689 | | 219 | | 31.8% | | 1.70 | | (1.38 to 2.11) | <0.001 | 1.70 | (1.37 to 2.10) | <0.001 |
| BCLP | 867 | | 287 | | 33.1% | | 1.81 | | (1.48 to 2.21) | <0.001 | 1.81 | (1.48 to 2.20) | <0.001 |
| *Adjusted for sex | |  | |  | |  | |  |  |  |  |  |  |
